# Healthcare Students' Flu Vaccine Uptake in the Last 5 Years and Future Vaccination Acceptance: Is There a Possible Association?

**DOI:** 10.34172/jrhs.2020.09

**Published:** 2020-03-29

**Authors:** Paola Cella, Matteo D’Angelo, Giulia Dallagiacoma, Sandro Provenzano, Omar Enzo Santangelo, Vincenza Gianfredi

**Affiliations:** ^1^Unit of Clinical Governance, Local Health Authority of Piacenza, Piacenza, Italy; ^2^Department of Medicine, University of Udine, Udine, Italy; ^3^Department of Public Health, Experimental and Forensic Medicine, University of Pavia, via Forlanini, Pavia, Italy; ^4^Department of Sciences for Health Promotion and Mother and Child Care ‘Giuseppe D’Alessandro’, University of Palermo, Palermo, Italy; ^5^Post-Graduate School of Hygiene and Preventive Medicine. Department of Experimental Medicine, University of Perugia, Perugia, Italy

**Keywords:** Survey and Questionnaires, Influenza Vaccines, Students, Health Occupations

## Abstract

**Background:** Despite the free-of-charge offer of influenza vaccines to at-risk subgroups, vaccine coverage remains low and far from the target, probably due to the false myths and misperceptions. We aimed to explore the healthcare students’ vaccination behavior and beliefs to find any association between vaccination uptake during the last 5 years and future vaccination acceptance.

**Study design:** A multicentre cross-sectional study.

**Methods:** From Oct 2017 to Nov 2018, the Italian healthcare students from 14 different universities in 2017/2018 were enrolled, through an online and anonymous questionnaire previously validated. Absolute and relative frequencies were calculated and Pearson's Chi-square test was used. A multinomial logistic regression model was performed. Results are expressed as relative Risk Ratio (RR) with 95% Confidence Intervals (95% CI). The level of significance chosen was P-value <0.05.

**Results:** A total of 3137 students were enrolled and 3131 questionnaires were analysed. 82.7% of the sample declared they had not received any flu vaccination during the last 5 years. Students who received flu vaccination 4 times or more during the last 5 years were more likely to do it again next year as well (95.1% vs 4.9%). The regression model showed that having received flu vaccination over the last 5 years was statistically associated with the intention of getting vaccinated during next season.

**Conclusions:** Frequency of flu vaccination is predictive for future acceptance among healthcare students. This association could have both implications for the organization of vaccination campaigns and improve educational strategies for this category of students.

## Introduction

Seasonal influenza is a viral infection, erroneously considered as a mild disease, that produces direct and indirect-related costs and social burden of public health concern ^1,2^.

The Centers for Disease Control and Prevention (CDC) recommends a yearly vaccination as the main tool in protecting against flu and its complications ^3^. Vaccination is one of the most cost-effective ways of avoiding disease, it currently prevents 2-3 million deaths a year, and a further 1.5 million could be avoided if global coverage of vaccinations improved^4^. Nevertheless, influenza immunization coverage remains universally low and far from the target. The reasons behind this are complex: WHO Strategic Advisory Group of Experts (SAGE) on vaccines identified the 3C model for explaining hesitancy. Complacency, convenience in accessing vaccines and confidence are key reasons underlying hesitancy ^5^.

Vaccine hesitancy is defined as the delay in acceptance or refusal of vaccination despite availability of vaccines ^6^. This represents a complex global problem that requires ongoing monitoring and, in times where vaccines are losing public confidence, strengthened efforts and resources to promote effective immunization strategies, train healthcare professionals and implement effective educational and communication programs^7,8^.

In Italy, official recommendations are issued by the Ministry of Health through the National Immunization Prevention Plan, a guidance document for immunization policies^9^, listing vaccines actively offered free of charge to the general population, to high-risk subjects, as well as to certain occupational groups^10-13^.

For example, flu vaccination is recommended for health care workers (HCWs) due to their occupational exposure and because they may act as vectors in the nosocomial transmission of influenza^14^. Furthermore, health care professionals are important role models for their patients; if they hesitate for any reason it can influence their patients’ willingness to vaccinate.

Healthcare students are the future healthcare workers, whose behavior will affect the health of their patients^15^. Moreover, due to the high level of close social interaction, frequent personal contacts between students, faculty and staff and their interface with the community, the university can become a focal point of epidemics for vaccine preventable diseases (VPDs) ^16^.

Despite the free of charge offer of influenza vaccines to medical students, flu vaccine coverage rates among this category are still far from the 75% target established by the Italian Ministry of Health.This suggests the importance to strengthen the recommendations in a population where high risk transmission does not consistently translate into high vaccination rates^17^.

Students’ perception, regarding vaccine-preventable diseases, influences their vaccination behavior. Misconceptions about influenza and influenza vaccine could be improved by education.

The current study explored healthcare students’ features, such as socio-demographic characteristics, knowledge, attitude and beliefs about vaccination, as determinants for immunization adherence and vaccination uptake during the last 5 years. The possible association between flu vaccination frequency and future uptake could have both implications for the organization of vaccination campaigns and improve educational strategies for this category of students.

## Methods

### Study design

This survey on healthcare students attending 14 Italian Universities was performed from Oct 2017 to Nov 2018. Each participant was interviewed via an anonymous, self-administered, and web-based questionnaire.

It was a multicenter cross-sectional study, approved by the Local Ethical Committee of the University of Perugia (*Comitato Universitario di Bioetica* ), Reference Number 2017-20R. The study was promoted by the Committee of Medical Residents of the Italian Society of Hygiene and Preventive Medicine.

The questionnaire administered was a validated survey, previously used for a study performed among Public Health Residents^18^. For this reason, a slight adaptation was carry out without changing the structure and the meaning of the items. All healthcare students, regardless of age and year of course, were eligible. Each resident in Public Health involved in the study was in charge to present the survey to the students of his/her university. Participation was anonymous and voluntarily. A Quick Response (QR) code redirecting to the online questionnaire was provided to the acceptant students.

### Statistical Analysis

The variable "age" was dichotomized in ≤23 years and > 23 years considering that the mean age was 23.41 years old and the responses about the degree course were aggregated into three groups according to the students' belonging to a certain category: medicine and surgery, nursing and other (which consists of all the other students of the healthcare professions who completed the questionnaire). According to the geographical area of origin, the answers were categorized into: “South and Islands” (if Bari, Messina, Naples, Palermo or Salerno), “Center” (if Ancona, L'Aquila, Perugia, Rome or Siena) and “North” (if Parma, Pavia, Turin or Udine). The answers to the question “Do you think your level of knowledge about vaccine-preventable diseases and related vaccinations is” were aggregated into two groups, "Good/excellent" and "Insufficient/sufficient/fair". The answers to the other questions were not aggregated. The absolute and relative frequencies were calculated for all qualitative variables and Pearson's Chi-square test (χ^2^) was used to analyze categorical variables.

A multinomial logistic regression model was used. The selected dependent variable was “How many times did you vaccinate against seasonal flu in the last 5 years? (Zero times was the reference, the answers "4" and "5 times" were aggregated)”. Each independent variable in the model was adjusted for all the other independent variables. Results are expressed as Relative Risk (RR) with 95% Confidence Intervals (95% CI). The level of significance chosen for statistical analysis was 0.05. Data were analyzed using statistical software STATA® version 14.

## Results

Overall, 3137 students compiled the survey, 6 of which had incorrectly filled the questionnaire and were therefore excluded from the analysis. As for the remaining 3131 subjects (age range 18-46), the variables gender, mean age, year of the degree course, flu vaccination during the previous 5 years and self-reported knowledge on VPDs and related vaccines are shown in Table 1.

**Table 1:** Sample characteristics (n=3131)

**Variables**	**Number**	**Percent**
Gender		
Female	2132	68.1
Male	999	31.9
Year of degree		
First	847	27.1
Second	672	21.5
Third	727	23.2
Fourth	153	4.9
Fifth	396	12.6
Sixth	216	6.9
Outside prescribed time	120	3.8
Did you get vaccinated, against seasonal flu, in the last 5 years?		
Never	2589	82.7
1 times in 5 years	171	5.5
2 times in 5 years	133	4.2
3 times in 5 years	54	1.7
4 times in 5 years	10	0.3
Every year	174	5.6
Self-reported knowledge on vaccines and related VPDs		
Good/Excellent	1349	43.1
Insufficient/sufficient/fair	1782	56.9

Importantly, 82.7% of the students declared they had never received the flu vaccination during the last 5 years, while 43.1% of the sample self-evaluated themselves as good or excellent experts on vaccines and related VPDs.

Demographic characteristics and vaccine knowledge, attitude and practices according to the number of flu vaccinations received during the last 5 years are presented in Table 2. Accordingly, the frequency of flu vaccination in the last 5 years was statistically associated with: age; degree course; geographic area; self-reported knowledge on VPDs and related vaccinations; intention to get flu vaccine for the next season; considering themselves a risk group; having recommended or intention to recommend flu vaccination and having taken part in a vaccination campaign; having received requests for clarification on vaccinations and lastly with opinion on mandatory vaccination. No association was found between the frequency of flu vaccination and gender and frequency of VPDs during the last 5 years. Students who got the flu vaccine almost every year (≥4 times) were more frequently female (65.2% vs 34.8, *P* = 0.182), older than 23 years (52.7% vs 47.3%, *P* <0.001), living in the north of Italy (41.3 vs 34.8 in South and Islands vs 23.9 in Center, *P* =0.018), and with a good/excellent self-declared knowledge on VPDs and related vaccines (56.5% vs 43.5%, *P* <0.001). Moreover, those who received the flu vaccination 4 times or more during the last 5 years were more prone to receive flu vaccination the next year, as well (95.1% vs 4.9%, *P* <0.001). Almost all participants considered themselves as a risk group (71.2%), recommended flu vaccination to family and the general population, but not to other HCWs, leaving to them the freedom to choose. Students who were vaccinated against flu 4 times or more were in favor of mandatory vaccinations both for HCWs (98.9%) and as a requisite for school access (97.3%), compared to 0.5% of the same group that was contrary to both. Lastly, only 4.9% of these students were directly involved in HCWs vaccination campaigns during their internships.

**Table 2:** Bivariate analysis showing the association between sample characteristics and number of flu vaccinations received in the last 5 years. Pearson's Chi-square test was used. Statistically significant results are in bold

**Variables**	**The number of vaccines against seasonal flu in the last 5 years**										
	**Never**		**1**		**2**		**3**		**≥4**		***P*** **value**
	**n**	**%**	**n**	**%**	**n**	**%**	**n**	**%**	**n**	**%**	
Gender											0.182
Female	1774	83.21	118	5.53	80	3.75	40	1.88	120	5.63	
Male	814	81.48	53	5.31	54	5.41	14	1.40	64	6.41	
Age (yr)											0.001
>23	996	79.62	89	7.11	50	4.00	29	2.32	87	6.95	
≤23	1592	84.68	82	4.36	84	4.47	25	1.33	97	5.16	
Degree Course											0.002
Medicine and Surgery	963	79.00	78	6.40	55	4.51	27	2.21	96	7.88	
Nursing	872	84.25	51	4.93	44	4.25	14	1.35	54	5.22	
Others	753	85.86	42	4.79	35	3.99	13	1.48	34	3.88	
Geographic area											0.018
South and Sicily	785	79.61	63	6.39	60	6.09	14	1.42	64	6.49	
Center	752	84.59	43	4.84	31	3.49	19	2.14	44	4.95	
North	1051	83.68	65	5.18	43	3.42	21	1.67	76	6.05	
VPDs and vaccinations knowledge											0.001
Good/excellent	1054	78.13	91	6.75	67	4.97	33	2.45	104	7.71	
Insufficient/Sufficient/Fair	1534	86.08	80	4.49	67	3.76	21	1.18	80	4.49	
VPDs during the last 5 years											0.153
Never	1386	83.49	81	4.88	61	3.67	31	1.87	101	6.08	
At least once	1087	81.36	80	5.99	70	5.24	21	1.57	78	5.84	
Intention to get flu vaccine next season											0.001
No	1949	95.63	40	1.96	31	1.52	9	0.44	9	0.44	
Yes	639	58.46	131	11.99	103	9.42	45	4.12	175	16.01	
Considering themselves as risk group											0.010
No	711	84.77	39	4.65	35	4.17	13	1.55	41	4.89	
I don’t know	300	87.21	15	4.36	17	4.94	0	0.00	12	3.49	
Yes	1577	80.95	117	6.01	82	4.21	41	2.10	131	6.72	
Flu vaccination recommended to patients last campaign											0.001
No	1270	91.24	45	3.23	38	2.73	9	0.65	30	2.16	
Based on my evaluation	353	73.08	40	8.28	27	5.59	19	3.93	44	9.11	
According ministerial indications	965	76.82	86	6.85	69	5.49	26	2.07	110	8.76	
Flu vaccination recommended to patients next campaign											0.001
No	875	91.82	30	3.15	24	2.52	9	0.94	15	1.57	
Based on my evaluation	452	78.75	39	6.79	29	5.05	17	2.96	37	6.45	
According ministerial indications	1261	78.62	102	6.36	81	5.05	28	1.75	132	8.23	
Flu vaccination recommended to HCW last campaign											0.001
No	2375	86.49	120	4.37	92	3.35	42	1.53	117	4.26	
Yes	213	55.32	51	13.25	42	10.91	12	3.12	67	17.40	
Participation in vaccination campaign											0.005
Yes	53	67.95	6	7.69	6	7.69	4	5.13	9	11.54	
No	2535	83.03	165	5.40	128	4.19	50	1.64	175	5.73	
Received requests on vaccinations											0.001
Yes	1333	79.06	116	6.88	85	5.04	36	2.14	116	6.88	
No	1255	86.85	55	3.81	49	3.39	18	1.25	68	4.71	
Opinion on mandatory vaccination for school access											0.008
Contrary	114	91.20	5	4.00	4	3.20	1	0.80	1	0.80	
Indifferent	169	91.35	5	2.70	6	3.24	1	0.54	4	2.16	
Favorable	2305	81.71	161	5.71	124	4.40	52	1.84	179	6.35	
Opinion regarding mandatory vaccination for HCW											0.001
Contrary	143	91.08	9	5.73	3	1.91	1	0.68	1	0.68	
Indifferent	225	93.75	7	2.92	6	2.50	1	0.42	1	0.42	
Favorable	2220	81.20	155	5.67	125	4.57	52	1.90	182	6.66	

Table 3 shows multinomial logistic regression and relative Risk Ratio (RR) with 95% Confidence Intervals (CI). Each independent variable is adjusted for all the other independent variables. In this section only the statistically significant results are reported (Table 3).

**Table 3:** Summary of multinomial logistic regression analysis and relative Risk Ratio (RR), based on 3131 observations, according to the number of flu vaccines received during the last 5 years. Each independent variable is adjusted for all the other independent variables. Statistically significant results are in bold.

**Variables**	**How many flu vaccinations in the last 5 years? (NEVER is the reference)**			
	**1**	**2**	**3**	**≥4**
	**RR (CI 95%)**	**RR (CI 95%)**	**RR (CI 95%)**	**RR (CI 95%)**
Gender				
Female	1.00	1.00	1.00	1.00
Male	0.90 (0.63, 1.30)	1.38 (0.94, 2.03)	0.70 (0.36, 1.34)	1.04 (0.73, 1.50)
Age				
As the unit increases	0.99 (0.63, 1.30)	0.98 (0.93, 1.04)	0.99 (0.92, 1.07)	1.00 (0.96, 1.05)
Degree Course				
Medicine and Surgery	1.00	1.00	1.00	1.00
Nursing	0.84 (0.56, 1.27)	0.72 (0.44, 1.16)	0.70 (0.35, 1.43)	0.76 (0.51, 1.15)
Other	1.05 (0.67, 1.63)	1.21 (0.74, 1.99)	0.93 (0.44, 1.97)	0.85 (0.53, 1.37)
Geographic area				
South and Sicily	1.00	1.00	1.00	1.00
Center	0.98 (0.63, 1.52)	0.72 (0.44, 1.16)	1.85 (0.88, 3.91)	1.06 (0.68, 1.67)
North	0.87 (0.58, 1.30)	0.55 (0.35, 0.87)	1.09 (0.52, 2.27)	0.94 (0.63, 1.41)
VPDs and vaccinations knowledge				
Good/excellent	1.00	1.00	1.00	1.00
Insufficient/Sufficient/Fair	0.86 (0.60, 1.21)	0.93 (0.63, 1.37)	0.68 (0.37, 1.23)	0.86 (0.61, 1.22)
Intention to get flu vaccine next season				
No	1.00	1.00	1.00	1.00
Yes	11.34 (7.45, 17.24)	10.71 (6.75, 7.01)	18.45 (8.37, 40.62)	49.14 (24.42, 98.90)
Considering themselves as risk group				
No	1.00	1.00	1.00	1.00
I don’t know	0.86 (0.45, 1.63)	1.06 (0.56, 1.99)	omitted	0.65 (0.32, 1.32)
Yes	0.85 (0.57, 1.28)	0.72 (0.46, 1.12)	0.85 (0.43, 1.67)	0.77 (0.51, 1.18)
Flu vaccination recommended to patients (last campaign)				
No	1.00	1.00	1.00	1.00
Based on my evaluation	2.28 (1.23, 4.23)	1.87 (0.94, 3.72)	7.07 (2.28, 21.94)	3.42 (1.78, 6.57)
According ministerial indications	1.98 (1.16, 3.37)	2.02 (1.13, 3.60)	4.68 (1.63, 13.42)	2.74 (1.58, 4.75)
Flu vaccination recommended to patients (next campaign)				
No	1.00	1.00	1.00	1.00
Based on my evaluation	0.62 (0.32, 1.22)	0.73 (0.35, 1.52)	0.34 (0.11, 1.05)	0.64 (0.29, 1.40)
According ministerial indications	0.45 (0.24, 0.81)	0.48 (0.25, 0.93)	0.17 (0.06, 0.49)	0.59 (0.29, 1.20)
Flu vaccination recommended to HCW (last campaign)				
No	1.00	1.00	1.00	1.00
Yes	3.14 (2.08, 4.75)	3.46 (2.18, 5.48)	1.75 (0.84, 3.67)	3.82 (2.54, 5.75)
Participation in vaccination campaign				
Yes	1.00	1.00	1.00	1.00
No	1.20 (0.46, 3.11)	1.03 (0.39, 2.71)	0.41 (0.12, 1.36)	0.85 (0.35, 2.05)
Received requests on vaccinations				
Yes	1.00	1.00	1.00	1.00
No	0.83 (0.57, 1.23)	1.01 (0.66, 1.54)	1.05 (0.54, 2.03)	1.37 (0.93, 2.01)
Opinion on mandatory vaccination (for school access)				
Contrary	1.00	1.00	1.00	1.00
Indifferent	1.38 (0.31, 6.03)	0.79 (0.17,3.66)	0.98 (0.04, 24.20)	2.46 (0.16, 37.98)
Favorable	1.75 (0.53, 5.80)	0.61 (0.16,2.32)	1.00 (0.08, 13.44)	1.68 (0.13, 21.59)
Opinion on mandatory vaccination (for HCW)				
Contrary	1.00	1.00	1.00	1.00
Indifferent	0.37 (0.11, 1.20)	1.18 (0.23, 6.03)	0.55 (0.02, 12.87)	0.32 (0.01, 7.77)
Favorable	0.31 (0.11, 0.81)	1.59 (0.36, 7.09)	0.98 (0.07, 13.01)	1.67 (0.13, 21.58)

Having received the flu vaccination only once during the last 5 years, compared to no vaccination at all, was statistically associated with: the intention to get flu vaccine during the next season; having recommended flu vaccination to patients or to family members during the last campaign, mainly based on one’s clinical evaluation but also according to ministerial indications; having recommended the flu vaccination to other HCWs. An inverse association was found with being in favor of mandatory vaccination for HCWs. Having received the flu vaccination only twice during the last 5 years, compared to having received no vaccination at all, was statistically associated with: the intention to get flu vaccine during next season (*P* ≤ 0.001); having recommended flu vaccination during last campaign (*P* ≤ 0.01), according to ministerial indications, and having recommended flu vaccination to HCWs (*P* ≤ 0.001). Having received the flu vaccination only three times during the last 5 years, compared to having received no vaccination at all, is statistically associated with: the intention to get flu vaccine during next season (*P* ≤ 0.001); having recommended flu vaccination during last campaign, mainly based on one’s clinical evaluation (*P* ≤ 0.001) but also according to ministerial indications (*P* ≤ 0.005).

An inverse association was found between having received the flu vaccination once (*P* ≤ 0.01), twice (*P* <0.05), or three times (*P* ≤ 0.001), the intention to recommend flu shot during next campaign, according to ministerial indications. Whilst, having received the flu vaccination at least four times during the last 5 years, compared to never having been vaccinated, is statistically associated with: the intention to get flu vaccine during next season (*P* ≤ 0.001); having recommended the vaccination during the last campaign, mainly based on one’s clinical evaluation (*P* ≤ 0.001) but also according to ministerial indications (p≤ 0.001); and having recommended flu vaccination to HCWs (*P* ≤ 0.001).

## Discussion

The current study confirmed the hypothesis that having been vaccinated against seasonal flu during the last 5 years predicts the future uptake of influenza vaccine among undergraduate healthcare students.

Particularly, the frequency of flu vaccination represents an important aspect in predicting flu vaccine acceptance: in relation to this, it was possible to investigate the impact of the factors considered both on the vaccination choice and in clinical practice during the survey period.

Considering the age of the students, table 2 shows a greater number of younger students (under 23 years) for the lowest vaccination frequency category with statistical significance compared to the remaining category by age. In addition, Table 3 reports a unitary increase (in years) of the age of students associated with a growing vaccination frequency, probably as proof of a greater awareness of the issue, in terms of adherence to the vaccination practice, supported by more years of study.

The reduced frequency of vaccination coverage among the youngest, in any case, is evocative of the need for an educational intervention in Italian universities. As a matter of fact, healthcare students must be considered an important part of the future public healthcare workforce and a significant educational resource for health information, including the importance of vaccinations^19-20^. In this perspective, medical schools play a crucial role in the achievement of higher flu vaccination coverage, according to the European Center for Disease Control (ECDC) requirements^19-20^. Indeed, the Italian National Immunization Prevention Plan 2017-2019 recommends flu vaccination to healthcare students as well ^21^. However, our results show a very low vaccination rate for all the degree courses considered (8.1% medicine vs. 5.2% nurses vs. 4% other courses). These critical data might be representative of a patch-worked implementation of healthcare students’ flu vaccination inscribed in a broader and more complex context represented by the educational offer of Italian universities, which undergoes significant variations from one university to another.

Only 43.1% of the sample consider their level of knowledge on vaccines and related VPDs as good/excellent with obviously more evidence for the students who have never been vaccinated. This is evocative of the lack of awareness and information about this topic, as documented in the international literature, due also to the heterogeneity of the educational offer from one university to another as already expressed ^22^.

It also emerges, concerning Table 3, how the RR values for the unit of reference are uniformly lower, to highlight that the adhesion to vaccination campaigns is associated with a more in-depth knowledge on VPDs and related vaccinations.

However, as already mentioned above, this study wants to highlight that, even though the core curriculum of public health programs covers the topic of flu vaccination, knowledge does not always guarantee a change in behavior^23^. In this regard, only 5.6% of the students underwent influenza vaccination every year, although the awareness of representing a professionally at risk category is uniformly spread among all vaccine frequency categories, just as none of them has suffered of more vaccine-related diseases in the last 5 years compared to another.

A 2014 study, investigating seasonal influenza vaccination coverage, attitudes and beliefs toward the vaccine among undergraduate students, found that only 20.6% of students received flu vaccination and nearly 50% of them had unsubstantiated fears about this practice ^16^. Furthermore, the decision of receiving a vaccination is often a habit rather than a professional and ethical value shared by all ^24-25^.

As confirmed by our study, only the students that had already been vaccinated in the previous 5 years showed the intention to receive the flu vaccination in the next vaccination campaign. Only 2.5% of the interviewed students was involved in the vaccination campaigns offered to health workers: this evokes the urgent need for intervention, and both the organization of multidisciplinary trainings and a transversal involvement of Public Health Departments could represent possible answers. As proof of this, a US study carried out during 9 years documented a rise in the medical residents’ vaccination coverage from 24% to 99% because of a well-conducted vaccination campaign^26^. These findings suggest that it is possible to get a very different vaccination compliance in different settings and the fact that medical residents, just like medical students, should probably be considered one of the most important HCW target groups for influenza vaccination implementation ^27^.

Healthcare students represent a special group because of their bridging role between academic knowledge, technical skills and compliance with patient counseling. Consequently, they have to be considered as a priority group to be actively involved in campaigns promoting vaccination as they represent “the future”. Furthermore, they include a core group that usually accepts vaccination, even without any adequate support from the academic institutions ^25,27^.

The vaccination choice also reflects the behavior of healthcare students in clinical practice. It is not surprising that an ever-increasing percentage of students, directly associated with the number of vaccinations carried out in the last 5 years, claim to have provided clarifications regarding the composition of the vaccine during the campaign, which is evocative of a greater attention and awareness on the subject.

Similarly, the answers given concerning the recommendations provided to relatives or other health professionals about the previous vaccination campaign, should be interpreted. Our results show an inverse association between planning on recommending the vaccination to family members, friends or the general population according to the ministerial recommendations and having received the flu vaccine less than 4 times during the past 5 years. The percentage of students recommending the vaccination according to the ministerial recommendations increases with the amount of times they were vaccinated against seasonal flu during the past 5 years. 48.7% among those who were never vaccinated, 59.7% among those who were only vaccinated once, 60.5% among those who were vaccinated twice, 51.8% among those who were vaccinated three times and 71.7% among those who were vaccinated 4 times or more.

An inverse association has also been observed between being in favor of mandatory vaccinations for HCWs and the subject’s frequency of seasonal flu vaccination during the past 5 years. This may be explained by the fact that this association results to be statistically significant only in the subgroup of students who were only vaccinated once: as a matter of fact, they might benefit from a mandatory vaccination policy more than students who tend to get vaccinated spontaneously almost every year.

However, about 80% of our sample stated that they never received the seasonal flu vaccine during the past 5 years: this shows how much the reference item chosen can affect and influence the analysis. Moreover, an inverse association between these factors was only observed when considering the future vaccination campaign, not the past ones. This might be due to the strong political debate against mandatory vaccinations that was going on in Italy at the time our survey was administered to the students^28^. A detailed analysis of regional differences of Health Sciences students' immunization behavior is reported in a companion paper ^29^.

This study presents the following limitations: at first, as a cross-sectional study, it did not allow to establish a causal relationship between variables; secondly, the use of a multiple-choice questions tool may have circumscribed the comprehension of such a complex phenomenon.

The anonymity of the questionnaire has however allowed limiting the potential social desirability bias. Moreover, being an online questionnaire with mandatory answers, missing data were avoided.

Lastly, the on-line administration is very economical and easy to use, allowing to collect a high number of responses.

## Conclusion

Having been vaccinated against seasonal flu during the last 5 years predicts the future uptake of influenza vaccine among undergraduate healthcare students.

Furthermore, although healthcare students should be receiving information about vaccinations within their curricula, it cannot be assumed that they will have positive attitudes and beliefs toward the seasonal influenza vaccine. Those variables were not exhaustively investigated in this study: further investigations may be required for a better understanding of this relationship. At last the need of improvement of university education to standardize the training offer for students in the healthcare area, could represent a possible answer to the criticality of the results emerged.

## Acknowledgements

The Authors would like to thank all members of the “Vaccine and Vaccine Hesitancy” working group of the Committee of Medical Resident of Italian Society of Hygiene and Preventive Medicine (Enrico Alagna, University of Palermo; Claudia Alessandroni, University of Roma Tor Vergata; Bruno Cosenza, University of Messina; Alessandro Cuda, University of Pavia; Francesco D’Aloisio, University of L’Aquila; Angelo D’ambrosio, University of Torino; Sara De Nitto, University of Bari; Francesca di Gaspare, University of Roma Tor Vergata; Leandro Gentile, University of Pavia; Giuseppe Ferrucci, University of Salerno; Francesco Mazzù, University of Messina; Pasquale Stefanizzi, University of Bari; Marina Di Vincenzo, University of Marche; Lucia Kundisova, University of Siena; Monica Navaro, University of Napoli Vanvitelli; Gianluca Voglino, University of Torino). Furthermore, we also thank all the students who anonymously and voluntary completed the questionnaire and researchers and professors who voluntary supported our research.

## Conflict of interest

None to declare.

## Funding

None.

## Highlights

- Immunization among healthcare students predicts future vaccination acceptance
- Healthcare students’ knowledge is not associated with a change in behavior
- A need of educational interventions in Italian Universities emerges
